# The Airways' Mechanical Stress in Lung Disease: Implications for COPD Pathophysiology and Treatment Evaluation

**DOI:** 10.1155/2019/3546056

**Published:** 2019-09-05

**Authors:** Pierachille Santus, Matteo Pecchiari, Francesco Tursi, Vincenzo Valenti, Marina Saad, Dejan Radovanovic

**Affiliations:** ^1^Department of Biomedical and Clinical Science (DIBIC), Università Degli Studi di Milano, Division of Respiratory Diseases, Ospedale L. Sacco—Polo Universitario, ASST Fatebenefratelli-Sacco, Milan, Italy; ^2^Department of Physiopathology and Transplantation, Università Degli Studi di Milano, Milan, Italy; ^3^Division of Respiratory Diseases, Ospedale Maggiore di Lodi, ASST Lodi, Lodi, Italy; ^4^Department of Health Bioscience, Università Degli Studi di Milano—Respiratory Unit, Policlinico di San Donato, IRCCS—San Donato Milanese, Milan, Italy

## Abstract

The airway epithelium stretches and relaxes during the normal respiratory cycle, and hyperventilation exaggerates this effect, resulting in changes in lung physiology. In fact, stretching of the airways influences lung function and the secretion of airway mediators, which in turn may cause a potentially injurious inflammatory response. This aim of the present narrative review was to illustrate the current evidence on the importance of mechanical stress in the pathophysiology of lung diseases with a particular focus on chronic obstructive pulmonary disease (COPD) and to discuss how this may influence pharmacological treatment strategies. Overall, treatment selection should be tailored to counterpart the effects of mechanical stress, which influences inflammation both in asthma and COPD. The most suitable treatment approach between a long-acting *β*2-agonists/long-acting antimuscarinic-agonist (LABA/LAMA) alone or with the addition of inhaled corticosteroids should be determined based on the underlying mechanism of inflammation. Noteworthy, the anti-inflammatory effects of the glycopyrronium/indacaterol combination on hyperinflation and mucociliary clearance may decrease the rate of COPD exacerbations, and it may synergistically improve bronchodilation with a double action on both the cyclic adenosine monophosphate (cAMP) and the acetylcholine pathways.

## 1. Introduction

The bronchial epithelium preserves lung homoeostasis and modulates airway inflammation; it plays a crucial role by producing both pro- and anti-inflammatory cytokines, affecting the migration of leucocytes in the airway submucosa and lumen and modulating the function of myofibroblasts and airway smooth muscle cells [1]. The airway epithelium stretches and relaxes during the normal respiratory cycle, and hyperventilation exaggerates this effect, resulting in changes to lung physiology. In fact, stretching of the airways influences lung function and the secretion of airway mediators, which, in turn, may cause a potentially injurious inflammatory response. Most experiments investigating this effect have been performed in static cultured cells, and evidence in dynamic models is scant [1].

This narrative review describes the current evidence on the importance of mechanical stress in the pathophysiology of lung diseases and chronic obstructive pulmonary disease (COPD) and discusses how this can influence treatment selection.

## 2. Airways' Mechanical Stress in Lung Diseases

The role of mechanical stimuli in airway remodelling has been hypothesized by Tschumperlin and Drazen more than a decade ago [2]. Unlike the common thinking of the period that considered airway remodelling to be consequent to the inflammatory milieu of the asthmatic setting, the authors indicated cellular stresses in the constricted airway, combined with a responsive cell population, as a plausible explanation of the mechanically responsive nature of the airway. The profibrotic environment produced by mechanical stress applied to the airway epithelial cells, in the absence of any inflammatory response, demonstrated the role of mechanical stimuli in airway remodelling [2]. Thomas et al. studied in vitro BEAS 2B cells, cyclically stretched using the Flexercell system and measured the levels of IL-8, RANTES protein, and RNA after variable elongations, rates, and duration of stretch [1]. In their study, the authors used a Ras homologous-associated kinase (Rho inhibitor) to assess the effect of blocking the downstream of integrin signaling. Immunofluorescent staining of paxillin was used to evaluate the effect of stretch on the distribution of focal contacts and the organisation of the actin cytoskeleton. The release of IL-8 induced by BEAS 2B cells was increased by cytokine stimulation and stretch, whereas RANTES levels in the cell supernatant decreased after stretch in a dose-, time-, and rate-dependent manner. A rate of elongation of 30% at 20 cycles/min for 24 h increased IL-8 levels by over 100% (*p* < 0.01). Conversely, blocking rho kinase using Y-27632 inhibited the stretching effect on IL-8 release by the BEAS 2B cells. The immunofluorescent staining showed the disassembly of focal adhesions during stretch, with a redistribution of paxillin to the perinuclear region. According to the study results, bronchial epithelial cell function is profoundly affected by stretching via the activation of rho kinases, and mechanotransduction in bronchial epithelial cells depends on a finely tuned coordination of a focal adhesion turnover (assembly and disassembly) [1].

In 2003, Tschumperlin et al. showed that bronchial epithelial cells increase the steady state level of mRNA for both ET-1 and ET-2, and they also increase the release of ET protein and TGF-*β*2 from a preformed cell-associated pool [3]. TGF-*β*2 and ET act both individually and synergistically to promote fibrotic protein synthesis in reporter fibroblasts. In fact, the elicited fibrotic protein synthesis in fibroblasts occurring after mechanical stress of the bronchial epithelial cells is significantly inhibited by combined treatment with ET receptor antagonists and a neutralizing antibody directed against TGF-*β*2. This study suggested that the bronchial epithelium contributed to the fibrotic environment of the airway wall and that the subepithelial fibrosis of the asthmatic airway wall may be caused by the mechanical forces accompanying bronchoconstriction.

Grainge et al. investigated the same phenomenon to evaluate the effects of repeated experimentally induced bronchoconstriction on airway structural changes in patients with asthma [4]. They randomly assigned 48 subjects with asthma to one of four inhalation challenge protocols involving a series of three challenges with one type of inhaled agent presented at 48-hour intervals. The two active challenges were with either a dust-mite allergen (which causes bronchoconstriction and eosinophilic inflammation) or methacholine (which causes bronchoconstriction without eosinophilic inflammation); the two control challenges (neither of which causes bronchoconstriction) were either saline alone or albuterol followed by methacholine (to control for non-bronchoconstrictor effects of methacholine). Bronchial biopsy specimens were obtained before challenges and 4 days after completion of the challenges. Overall, allergen and methacholine immediately induced similar levels of bronchoconstriction. Eosinophilic inflammation of the airways increased only in the allergen group, whereas both the allergen and the methacholine groups showed significant airway remodelling not seen in the two control groups. Subepithelial collagen-band thickness increased by a median of 2.17 *μ*m in the allergen group (interquartile range (IQR): 0.70–3.67) and 1.94 *μ*m in the methacholine group (IQR: 0.37–3.24; *p* < 0.001 challenge groups vs. control groups); periodic acid-Schiff staining of epithelium (mucus glands) also increased, by a median of 2.17 percentage points in the allergen group (IQR: 1.03–4.77) and 2.13 percentage points in the methacholine group (IQR: 1.14–7.96; *p*=0.003 for the comparison with controls). There were no significant differences between the allergen and methacholine groups. This study suggested that bronchoconstriction induces epithelial stress and initiates a tissue response that leads to structural airway changes. The prevention of bronchoconstriction itself should represent therefore an important aim of asthma management, since repeated epithelial stress may cause airways' remodelling.

Noteworthy, airway inflammation also characterizes COPD [5], as well as heavy smokers who do not develop airflow obstruction. However, in the latter group, the inflammatory response is qualitatively similar to the one seen in smoking-related COPD. A few studies showed a relationship between the severity of airflow obstruction and the degree of inflammation, supporting the hypothesis that airway inflammation plays an important role in COPD. The airway wall exposed to inflammatory stimuli shows increased levels of CD8+ T cells, macrophages, neutrophils, and mast cells, whereas B cells are increased in more severe COPD stages. The main site of airflow obstruction in COPD is represented by the small airways, the cytology of which is difficult to access. However, the pattern of airway inflammation seen in larger airways, which are easily accessible to bronchoscopic biopsy techniques, is essentially similar to the one found in the smaller airways. Nevertheless, it is difficult to precisely determine which are the most important cells, cytokines and mediators involved in the process. Ultimately, understanding which pathway is important will depend on intervention studies attempting to associate changes in inflammation with clinical benefit.

The role of bronchodilators is essential in the treatment of COPD, and these drugs could play a primary role in terms of airways mechanical stress modulation. Recently, Cazzola et al. characterized the pharmacological interaction between two bronchodilators as glycopyrronium bromide and indacaterol fumarate and identified the mechanisms leading to the bronchorelaxant effect of this interaction [6]. The interaction between glycopyrronium and indacaterol on the contractile tone of medium and small human isolated bronchi was assessed by the Bliss Independence approach. Glycopyrronium + indacaterol synergistically inhibited the bronchial tone (medium bronchi: +32.51% ± 7.86%; small bronchi: +28.46% ± 5.35%; *p* < 0.05 vs additive effect). The maximal effect was reached 140 min post-administration. A significant synergistic effect was observed during 9 h after administration on the cholinergic tone but not on the histaminergic contractility (*p* < 0.05). Coadministration of glycopyrronium and indacaterol reduced acetylcholine release from the epithelium but not from bronchi and enhanced cAMP levels in bronchi and epithelial cells (*p* < 0.05 vs control). This effect was inhibited by the selective KCa(++) channel blocker, iberiotoxin. The role of cAMP-dependent pathway was confirmed by the synergistic effect elicited by the adenylate cyclase activator forskolin on glycopyrronium (*p* < 0.05 vs additive effect), but not on indacaterol (*p* > 0.05 vs additive effect), with regard to the bronchial relaxant response and cAMP increase. These findings suggested that glycopyrronium/indacaterol coadministration leads to a synergistic improvement of bronchodilation by increasing cAMP concentrations in both airway smooth muscle and bronchial epithelium and by decreasing acetylcholine release from the epithelium.

## 3. Clinical Evidence

Small airways play an important role in the pathogenesis of COPD, and their functional evaluation relies on specific lung functional tests, known from many years [7]. Taking in account these pathophysiological evidence, it should be underlined that further damage and substantial inflammatory reactions may occur when small airway collapse is enhanced by increased surface tension [8], thus supporting the hypothesis that the cyclic opening and closing of small airways during tidal breathing causes lung injury in man [9]. More recently, Pecchiari et al. demonstrated that airway closure involves a substantially greater fraction of the resting tidal volume in a population of severe and very severe COPD patients. Therefore, cyclic opening and closure could perpetrate the damage to normal airways as it happens in already altered airways [10].

A landmark trial has recently investigated whether destruction of the terminal and transitional bronchioles (the first generation of respiratory airways) occurs before or in parallel with emphysematous tissue destruction [11]. In this cross-sectional analysis, a novel multiresolution CT imaging protocol was applied to tissue samples of smokers with normal lung function (controls, *n* = 10) and patients with mild COPD (Global Initiative for Chronic Obstructive Lung Disease (GOLD) stage 1, *n* = 10), moderate COPD (GOLD 2, *n* = 8), or very severe COPD (GOLD 4, *n* = 6), with centrilobular emphysema. Patients with GOLD 1 or GOLD 2 COPD and smokers with normal lung function had undergone lobectomy and pneumonectomy, and patients with GOLD 4 COPD had undergone lung transplantation. Lung tissue samples were used for stereological assessment of the number and morphology of terminal and transitional bronchioles, airspace size (mean linear intercept), and alveolar surface area. The 34 lung specimens provided 262 lung samples. Compared with control smokers, the number of terminal bronchioles decreased by 40% and 43% in patients with GOLD 1 and GOLD 2 COPD (*p*=0.014 and *p*=0.036, respectively), whereas the number of transitional bronchioles decreased by 56% and 59% in patients with GOLD 1 and GOLD 2 COPD, respectively (both *p*=0.0001). The alveolar surface area decreased by 33% and 45% in patients with GOLD 1 and GOLD 2 COPD (*p*=0.019 and *p*=0.0021, respectively). In several cases, significant loss of terminal and transitional bronchioles was noted also in case of normal alveolar surface area, and all these pathological changes correlated with lung function decline. The remaining small airways showed thickened walls and narrowed lumens, becoming more and more obstructed with increasing COPD GOLD stage. The authors concluded that small airways disease is a pathological feature in mild and moderate COPD. Importantly, this study emphasizes that early disease-modifying interventions might be necessary for patients with mild or moderate COPD.

Barnes et al. tested the hypothesis that inhaled combined long-acting *β*2-agonist (salmeterol) and corticosteroid (fluticasone propionate) may reduce inflammation [5]. Bronchial biopsies and induced sputum from 140 current and former smokers with moderate-to-severe disease were randomized in a 13-week double-blind study to placebo (*n* = 73) or salmeterol/fluticasone propionate 50/500 mg (*n* = 67) twice daily and repeated at 12 weeks (biopsy) or at 8 and 13 weeks (sputum). After adjustment for multiplicity, the active treatment group was compared with the placebo group for median change from baseline in the numbers of biopsy CD8+ and CD68+ cells/mm^2^ and sputum neutrophils. Combination therapy was associated with reduced CD8+ cells in biopsy (−118 cells/mm^2^; 95% confidence interval (CI): −209 to −42; *p*=0.02), a 36% difference over placebo (*p*=0.001). CD68+ cells were unaffected by the combination treatment. Sputum differential (but not total) neutrophils decreased progressively, and at week 13, they were significantly reduced with the combination treatment (median treatment difference: 8.5%; 95% CI: 1.75–15.25%; *p*=0.04). The combination therapy also significantly reduced biopsy CD45+ and CD4+ cells and cells expressing genes for tumor necrosis factor-alpha and IFN-gamma and sputum total eosinophils (all *p* ≤ 0.03). These anti-inflammatory effects were accompanied by a 173-ml improvement in pre-bronchodilator forced expiratory volume in 1 s (FEV1; 95% CI: 104–242; *p* < 0.001). Overall, the combination of salmeterol and fluticasone propionate showed a broad spectrum of anti-inflammatory effects in both current and former smokers with COPD, which may contribute to its clinical efficacy.

Pecchiari et al. investigated the acute effects of tiotropium (18 *μ*g) and indacaterol (150 *μ*g) on closing volume (CV) and ventilation inhomogeneity in 51 stable patients with moderate to very severe COPD [12]. Patients underwent body plethysmography, arterial blood gas analysis, negative expiratory pressure to discriminate the presence of tidal expiratory flow limitation (EFL), dyspnea assessment, and simultaneous recording of single-breath nitrogen washout (SBN_2_) test and transpulmonary pressure-volume curve (PL-V), before and 1 h after drug administration. Except for vital and inspiratory capacity, the effects produced by indacaterol on each variable did not differ from those caused by tiotropium. Bronchodilators significantly decreased the slope of phase III and CV (−5 ± 4 and −2.5 ± 2.1%, respectively, both *p* < 0.001), with an increase in both the slope and the height of phase IV and of the anatomical dead space. Arterial oxygen pressure and saturation significantly improved (3 ± 3 mmHg and 2 ± 2%, respectively, both *p* < 0.001), and their changes negatively correlated with those of phase III slope (*r* = −0.659 and *r* = −0.454, respectively, both *p* < 0.01). The vital capacity increased substantially, but the PL-V/VC curve above CV was unaffected. The authors concluded that both muscarinic antagonists and *β*-adrenergic agonists provide airways bronchodilation by improving the mechanical properties of peripheral airways and the extent of their closure, with minor effects on critical closing pressures, and positively affecting gas exchange. Previously, Santus et al. evaluated the effect of a 4-week treatment with two different bronchodilators of common practice in COPD treatment, on the production of reactive oxygen species (ROS), such as superoxide anions, and of leukotriene B_4_ (LTB_4_) by peripheral blood neutrophils obtained from COPD patients [13]. They enrolled 24 COPD outpatients, who were randomized to receive either formoterol (12 *μ*g twice daily) or tiotropium (18 *μ*g once daily). Peripheral blood neutrophils were obtained at the start and at the end of the treatment, and production of superoxide anions and of LTB_4_ was evaluated. The authors demonstrated a decrease in the unstimulated production of superoxide by isolated neutrophils in both groups and a modulation of LTB_4_ production for tiotropium only, whereas formoterol caused an increased production of superoxide in response to the chemotactic factor N-formyl-l-methionyl-l-leucyl-phenylalanine (fMLP), when compared with values obtained before treatment [13]. These data sustained the notion that, in COPD patients, tiotropium has a better anti-inflammatory activity profile compared with formoterol. The latter results sustain the evidence that tiotropium can modulate the Ach-mediated LTB_4_ release, thus indirectly influencing the granulocyte chemotactic activity and in human airways [14].

The potential ancillary non-bronchodilator properties of tiotropium have been extensively investigated in vitro and in animal models [15, 16]. In fact, tiotropium demonstrated a potential role in modulating the neutrophilic airway inflammation and neutrophil adhesion by controlling the release of TGF-*β* [17]; moreover, tiotropium was shown to modulate the pro-inflammatory effects of IL-17 induced release of IL-8 in bronchial epithelial cells [18], to have a positive effect on lung fibroblast proliferation [19], and to modulate airway inflammation in a model of rhinovirus infection by inhibiting the activation of nuclear factor (*κ*)B proteins [20]. Tiotropium may also counteract to some extent the nonneuronal acetylcholine mediated production of Th17 cells in patients with COPD [21] and inhibit the IL-13-induced goblet cells metaplasia in healthy airway epithelial cells [22].

Santus and coworkers recently tested whether LABAs can affect the exhaled alveolar fraction of nitric oxide (CANO), an indirect inflammatory biomarker of the peripheral lung, correlating CANO with lung mechanics in patients with COPD [23]. The study was a two-center, randomized, double-blind, crossover study including 45 COPD patients with moderate-to-severe airflow obstruction after a period of pharmacological washout. In the study, multiflow exhaled fraction of nitric oxide (FeNO), plethysmography, lung diffusion capacity for carbon monoxide (DLCO), SBN_2_ test, and dyspnea were measured in a crossover manner at baseline and 30, 60, and 180 min after the administration of salmeterol (Sal) or formoterol fumarate (FF). At baseline, CANO correlated with airway resistances (*r* = 0.422), residual volume/total lung capacity (RV/TLC; *r* = 0.375), transfer factor (KCO; *r* = −0.463), and FEV_1_ (*r* = −0.375, all *p* < 0.01). After LABA administration, FeNO was significantly reduced at 180 min, without differences between FF and Sal. Moreover, the authors reported a significant reduction in CANO at 60 and 180 min compared with baseline for both FF and Sal (*p* < 0.01 and *p* < 0.05, respectively), and the changes in CANO being correlated with improvements in VC (*r* = −44; *p* < 0.001) and RV/TLC (*r* = 0.56; *p* < 0.001) but not FEV_1_. Importantly, the levels of CANO appeared to be directly associated to the magnitude of peripheral airway dysfunction in COPD, since LABA administration reduced the CANO levels and the reduction was associated with improvements in functional parameters reflecting air trapping.

At present, the available clinical results support the possibility that the effects of LABA/LAMA combinations on hyperinflation and mucociliary clearance may contribute to decreased COPD exacerbations [24]. Although preclinical studies suggest LABAs and LAMAs have anti-inflammatory effects, such effects have not yet been demonstrated in patients with COPD.

However, preliminary in vitro findings suggest that the specific LABA/LAMA combination of glycopyrronium/indacaterol may synergistically improve bronchodilation by increasing cAMP concentrations in both airway smooth muscle and bronchial epithelium and by decreasing acetylcholine release from the epithelium [6].

## 4. Implications for Clinical Practice

**Figure 1:**
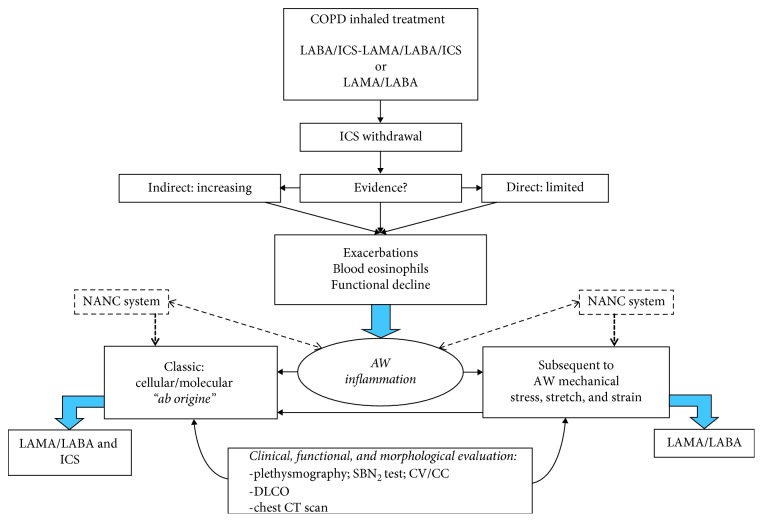
Inhalation treatment selection in COPD according to the underlying mechanism of inflammation. AW: airway flow.

According to the available evidence, a careful evaluation on the pathophysiological mechanisms leading to inflammation in COPD may have a major role in the selection of treatment (Figure 1).

Briefly, clinicians should look for direct and/or indirect evidence of inflammation (blood eosinophil counts, disease exacerbations, and functional decline) and of its origin, either typical (cellular and/or molecular, “*aborigine*”) or secondary to mechanical stress (“stretch and strain”), and according to the findings, introduce the proper treatment with a combination of LABA/LAMA with or without inhaled corticosteroids.

The noncholinergic nonadrenergic system (NANC) should be also taken in consideration. NANC, particularly as an additional branch of the cholinergic system, is the nonneuronal cholinergic system expressed in nearly all cell types present in the bronchial tree, with specific regards for distal airways. When NANC is activated, it contributes to an increased cholinergic tone in the respiratory system inducing different pathophysiological processes, such as inflammation, remodelling, chronic cough, and mucus hypersecretion [25]. In clinical practice, in view of the aforementioned evidence, the application of a selective LAMA as the initial treatment choice in patients with COPD could thus exert some positive non-bronchodilator effects, acting on neuronal and nonneuronal Ach-mediated inflammatory pathways. This may have an impact on the regional inflammatory environment, with additive beneficial effects on the modulation of the “traditional” inflammatory pathways together with the proven bronchorelaxant effect in patients with COPD.

Additionally, a global evaluation including clinical, functional, and morphological assessment by means of complete lung function tests, SBN_2_ test, DLCO, and chest CT scan should be performed before the therapeutic choice. The flow chart does not consider initial and less severe COPD stages, where only a single bronchodilator may be indicated.

Indeed, in COPD patients, it is widely accepted that exacerbations, eosinophilia, and functional decline ultimately leading to inflammation. However, the underlying mechanism that contributes most to the inflammatory process can be either molecular or secondary to mechanical stress. In the latter case, inhaled corticosteroids may not have a major role in the treatment paradigm; on the other hand, therapy with a LABA/LAMA combination will appear more suitable. Patients on long-term treatment with ICS-containing regimens should thus be always evaluated for ICS de-escalation or withdrawal. To date, the direct evidence of the clinical and pathophysiological effects of ICS withdrawal in patients with COPD is limited but consistent, and this has led to the publication of numerous de-escalating algorithms [26–29] The indirect proof that ICS withdrawal did not increase the exacerbation rate was shown by the subgroup analysis of a recently published real life, noninterventional, longitudinal prospective cohort study conducted in primary and secondary care patients with COPD [30]; these results were confirmed in a subgroup analysis of the CRYSTAL study, a pragmatic trial that shows that symptomatic patients with moderate COPD switched from a treatment with LABA/ICS to indacaterol/glycopyrronium which demonstrated a significant increase in FEV_1_ (71 mL, *p* < 0001) and TDI score (+1.10 units, *p* < 0.0001) [31]. In 2014, Rossi et al. performed a six-month randomized double-blind double-dummy parallel group study in which patients with moderate COPD without exacerbations in the previous year were switched from salmeterol/fluticasone 50/500 mcg BID to indacaterol 150 mcg, without any sign of loss of efficacy in the indacaterol-treated group [32]. Comparable results were observed in a six-month real life study in which patients with <2 exacerbations in the previous year and moderate COPD underwent a de-escalation therapy from ICS-containing regimens to long-acting bronchodilators only [33]. More recently, Kaplan and colleagues demonstrated that a direct switch to indacaterol/glycopyrronium in symptomatic patients with moderate-to-severe COPD despite treatment with tiotropium alone or the combination of salmeterol/fluticasone was safe and provided clinically significant improvements in lung function, quality of life, and disease-related symptoms [34].

The WISDOM study [35] first demonstrated that ICS withdrawal in patients with severe COPD and at least one exacerbation in the previous year did not increase the exacerbation frequency in patients treated with the association of indacaterol/glycopyrronium. A post hoc subgroup analysis of the trial found that patients with <2% of blood eosinophils were those that benefited most of the ICS withdrawal in terms of exacerbation risk [36]. These results were prospectively confirmed in the SUNSET study [37], a 26-week randomized double-blind triple-dummy clinical trial; patients with COPD at low risk of exacerbations in triple therapy with tiotropium and salmeterol/fluticasone were randomized to directly switch to indacaterol/glycopyrronium or to continue the triple therapy. Again, patients with baseline ≥300 blood eosinophils/*μ*l presented greater lung function loss and higher exacerbation risk. We therefore suggest that, upon a meticulous assessment of the clinical history and confirmation of the diagnosis, in all COPD patients, independent of the degree of airflow obstruction, that show clinical stability in the last two years and without elevated blood eosinophils (i.e. ≥400 cells/*μ*l), the withdrawal of ICS and the initiation of a LABA/LAMA treatment should always be considered. To date, high quality and direct evidence suggesting if twice daily or once daily dosing regimens for approved fixed dose LABA/LAMA combinations is lacking. In terms of efficacy, indirect comparisons performed by means of systematic reviews and meta-analyses did not point out any clinical significant difference [38, 39]. A “gradient of effectiveness” of LABA/LAMA combinations in terms of trough FEV_1_ has been postulated by Calzetta et al. in their meta-analysis [38], with the first place shared by two different dosing regimens (i.e., once and twice daily). Taking in consideration the great impact of adherence to inhaled treatment on patient-related outcomes [40], once-daily dosing regimens had been advocated to favor adherence among patients with COPD; however, this hypothesis is only weakly sustained by clinical evidence [41, 42]; accordingly, twice-daily LABA/LAMA combinations should sustain an increased symptom control through night-time and on awakening [43], but direct and conclusive comparator studies to assess any difference in dosing regimens are currently lacking.

A proper clinical and pathophysiological assessment of disease and patient's characteristics should therefore always serve as the guidance for treatment selection.

## 5. Conclusion

It has been shown that mechanical stress plays an important role in the pathophysiology of lung diseases and COPD. Thus, treatment selection should be tailored to counterpart the effects of mechanical stress, which influences inflammation in COPD and asthma. The most suitable treatment approach between a LABA/LAMA alone or with the addition of inhaled corticosteroids should be determined based on the underlying mechanism of inflammation. Noteworthy, the anti-inflammatory effects of the glycopyrronium/indacaterol combination on hyperinflation and mucociliary clearance may decrease the rate of COPD exacerbations, and it may synergistically improve bronchodilation with a double action on both the cAMP and the acetylcholine pathways. Moreover, evidence suggests that a tailored therapeutic approach with bronchodilators may be relevant also in asthma, in order to prevent the remodelling of the asthmatic airway wall caused by the mechanical stress associated with bronchoconstriction.

## Abbreviations

CANO: Exhaled alveolar fraction of nitric oxide
COPD: Chronic obstructive pulmonary disease
CV: Closing volume
DLCO: Lung diffusion capacity for carbon monoxide
EFL: Expiratory flow limitation
FEV_1_: Forced expiratory volume in one second
FeNO: Exhaled fraction of nitric oxide
FF: Formoterol fumarate
LTB_4_: Leukotriene B_4_
PL-V: Transpulmonary pressure-volume curve
ROS: Reactive oxygen species
RV: Residual volume
Sal: Salmeterol
SBN_2_: Single-breath nitrogen washout
TLC: Total lung capacity
VC: Vital capacity.
